# (*Z*)-Isobutyl 2-benzamido-3-(4-chloro­phen­yl)acrylate

**DOI:** 10.1107/S1600536809043931

**Published:** 2009-10-28

**Authors:** Gui-Fa Su, Zhong-Chang Wang, Wan-Yun Huang, Zhi-Xin Wang, Zi-Lu Chen

**Affiliations:** a College of Chemistry and Chemical Engineering, Guangxi Normal University, Guilin 541004, People’s Republic of China

## Abstract

The title compound, C_20_H_20_ClNO_3_, is a α-amino acid derivative which displays a *Z* configuration about the C=C double bond. The dihedral angle betwen the aromatic rings is 87.75 (12)°. The mol­ecular conformation is stabilized by an intra­molecular C—H⋯N hydrogen bond. In the crystal structure, centrosymmetrically related mol­ecules inter­act through inter­molecular C—H⋯O hydrogen-bond inter­actions, forming dimers. The dimers are further linked into chains parallel to the *a* axis by N—H⋯O hydrogen bonds. The methyl groups of the isopropyl group are disordered over two positions with occupancy factors of 0.5.

## Related literature

For the synthesis and crystal structure of related compounds, see: Jiménez *et al.* (2000 ▶); Peggion *et al.* (2003 ▶).
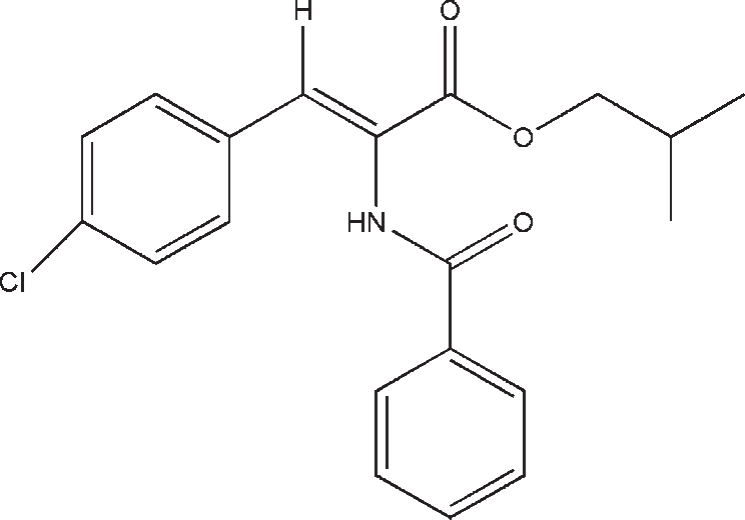


## Experimental

### 

#### Crystal data

C_20_H_20_ClNO_3_
*M* *_r_* = 357.82
Triclinic, 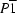

*a* = 5.0179 (10) Å
*b* = 12.581 (2) Å
*c* = 16.293 (3) Å
α = 67.623 (11)°
β = 83.991 (15)°
γ = 79.548 (14)°
*V* = 934.6 (3) Å^3^
*Z* = 2
Mo *K*α radiation
μ = 0.22 mm^−1^
*T* = 293 K
0.50 × 0.40 × 0.25 mm

#### Data collection

Rigaku AFC-7S Mercury diffractometer
Absorption correction: multi-scan (*REQAB*; Jacobson, 1998 ▶) *T*_min_ = 0.897, *T*_max_ = 0.946
9082 measured reflections
3380 independent reflections
2224 reflections with *I* > 2σ(*I*)
*R*_int_ = 0.036

#### Refinement

*R*[*F* ^2^ > 2σ(*F* ^2^)] = 0.073
*wR*(*F* ^2^) = 0.213
*S* = 1.10
3380 reflections
231 parameters
5 restraints
H atoms treated by a mixture of independent and constrained refinement
Δρ_max_ = 0.40 e Å^−3^
Δρ_min_ = −0.37 e Å^−3^

### 

Data collection: *CrystalClear* (Rigaku/MSC, 2000 ▶); cell refinement: *CrystalClear*; data reduction: *CrystalStructure* (Rigaku/MSC, 2004 ▶); program(s) used to solve structure: *SHELXS97* (Sheldrick, 2008 ▶); program(s) used to refine structure: *SHELXL97* (Sheldrick, 2008 ▶); molecular graphics: *SHELXTL* (Sheldrick, 2008 ▶); software used to prepare material for publication: *SHELXTL*.

## Supplementary Material

Crystal structure: contains datablocks I, global. DOI: 10.1107/S1600536809043931/rz2372sup1.cif

Structure factors: contains datablocks I. DOI: 10.1107/S1600536809043931/rz2372Isup2.hkl

Additional supplementary materials:  crystallographic information; 3D view; checkCIF report

## Figures and Tables

**Table 1:** Hydrogen-bond geometry (Å, °)

*D*—H⋯*A*	*D*—H	H⋯*A*	*D*⋯*A*	*D*—H⋯*A*
C3—H3⋯O1^i^	0.93	2.43	3.299 (4)	155
N1—H1⋯O3^ii^	0.86	2.07	2.916 (3)	169
C9—H9⋯N1	0.93	2.55	3.103 (4)	119

Symmetry codes: (i) 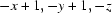; (ii) 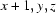.

**Fig. 1.**
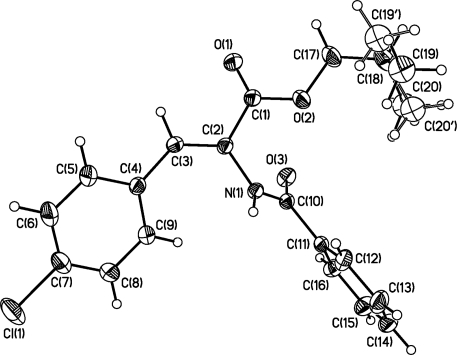
The molecular structure of the title compound, showing 20% probability displacement ellipsoids.

**Fig. 2.**
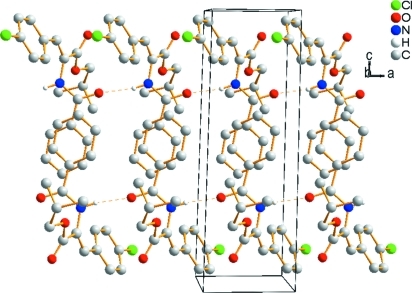
Partial packing diagram of the title compound showing the formation of chains parallel to the a axis. Intermolecular hydrogen bonds are shown as dashed lines. Hydrogen atoms not involved in hydrogen bonding are omitted for clarity.

**Fig. 3.**
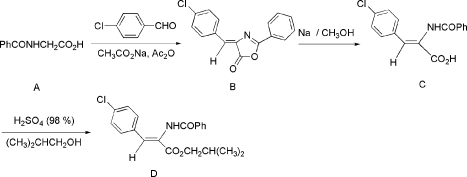
Synthesis of the title compound.

## supplementary crystallographic information

### Comment

As part of a study on the effect of the conformationally restricted molecular substitution on the crystal structures of biologically important class of compounds, we report herein the crystal structure of the title compound. Small and medium α-amino acids are generally highly flexible molecules that exist in solution in a dynamic equilibrium of interchanging conformations. As a consequence, most natural α-amino acids with physiological activity cannot be used for therapeutic purposes so we developed the introduction of conformational constrains. In comparison with native α-amino acids, side chain restricted analogues usually display more favorable pharmacological properties (Jiménez *et al.*, 2000; Peggion *et al.*, 2003).

The molecule of the title compound (Fig. 1) displays a *Z* configuration about the C2C3 double bond. The molecular conformation is enforced by an intramolecular C—H···N hydrogen bond (Table 1). The C19 and C20 methyl groups of the isopropyl group are disordered over two positions with occupancy factors of 0.5. The dihedral angle formed by the aromatic rings is 87.75 (12)°. In the crystal packing, centrosymmetrically related molecules are linked into dimers by intermolecular C—H···O hydrogen bonds. The dimers are further connected by N—H···O hydrogen bonds to form chains parallel to the *a* axis (Fig. 2).

### Experimental

Compound B (Fig. 3): to a 100 ml round-bottomed flask was added 1.4 g (1.18 ml, 0.01 mol) of redistilled 4-chlorobenzaldehyde, 1.79 g (0.01 mol) of benzoylglycine, 3.1 g (2.8 ml, 0.03 mol) of acetic anhydride and 0.82 g (0.01 mol) of anhydrous sodium acetate, and the mixture was heated on an electric hotplace with constant shaking. Once liquefied completely, the round-bottomed flask was transferred to a water bath and heated at 100 °C for 2 h, then 16 ml of ethanol was added slowly to the flask and the mixture allowed to stand overnight. The crystalline product was filtered with suction, washed twice with 25 ml of ice-cold alcohol and twice with 25 ml of boiling water and dried to afford 1.91 g of pure compound B (yield 64%).

Compound C (Fig. 3): to a 0.1% solution of sodium methoxide in absolute methanol (40 ml) was added 2.1 g of compound B (3.52 mmol). The mixture was heated to 75 °C under vigorously stirring until TLC analysis indicated that the starting material had disappeared (about 2 h). The product was collected by vacuum filtration and washed with small portions of cold methanol to afford 2.16 g of compound C as a white solid (yield 92%).

Title compound (D, Fig. 3): to a 100 ml round-bottomed flask was added 0.303 g (1.0 mmol) of compound C, 9.9 g (10 ml, 0.13 mol) of redistilled isobutanol, 10 ml of redistilled cyclohexane and 2 ml of concentrated sulfuric acid under stirring. The mixture was refluxed 4 h with stirring, then cooled and the product extracted with chloroform (2× 15 ml). The combined organic layer was dried over MgSO_4_, filtered and the solvent removed under reduced pressure to afford 0.293 g of the title compound as a white solid (yield 81%). Crystals suitable for X-ray analysis were obtained by slow evaporation of a dichloromethane-ethanol solution (1:2 *v*/*v*). IR 3837, 3745, 3648, 3165, 1737. ^1^H NMR (DMSO-d_6_, 500 MHz) δ 0.85 (d, 6H, J=6.75 Hz); 1.86 (m, 1H); 3.91 (d, 2H, J=6.4 Hz); 7.38 (s, 1H); 7.46 (d, 2H, J=8.5 Hz); 7.49–7.53 (m, 2H); 7.57–7.59 (m, 1H); 7.68 (d, 2H, J=8.5 Hz); 7.93 (d, 2H, J=7.1 Hz); 10.0 (s, 1H). ^13^C NMR (DMSO-d_6_, 125.8 MHz) δ 18.7, 27.2, 70.7, 127.4, 127.5, 128.4, 128.6, 131.3, 131.6, 131.8, 132.3, 133.2, 133.9, 164.8, 166.4.

### Refinement

The H atom on C18 was located in a difference Fourier map and refined isotropically. Other H atom were positioned geometrically and included in the refinement in the riding-model approximation, with C—H = 0.93–0.97 Å, N—H = 0.86 Å, and with *U*ĩso~(H) = 1.5*U*~eq~(C, N). The methyl groups C19 and C20 of the isopropyl group are disordered over two positions with occupancy factors of 0.5 and were refined isotropically. The C—C distances within the isopropyl group were restrained to be 1.54 (1) Å.

### Crystal data

**Table:**

C_20_H_20_ClNO_3_	*Z* = 2
*M**_r_* = 357.82	*F*(000) = 376
Triclinic, *P*1	*D*_x_ = 1.271 Mg m^−^^3^
Hall symbol: -P 1	Mo *K*α radiation, λ = 0.71070 Å
*a* = 5.0179 (10) Å	Cell parameters from 2536 reflections
*b* = 12.581 (2) Å	θ = 3.3–25.3°
*c* = 16.293 (3) Å	µ = 0.22 mm^−^^1^
α = 67.623 (11)°	*T* = 293 K
β = 83.991 (15)°	Block, colourless
γ = 79.548 (14)°	0.50 × 0.40 × 0.25 mm
*V* = 934.6 (3) Å^3^	

### Data collection

**Table:**

Rigaku AFC-7S Mercury diffractometer	3380 independent reflections
Radiation source: fine-focus sealed tube	2224 reflections with *I* > 2σ(*I*)
graphite	*R*_int_ = 0.036
Detector resolution: 7.31 pixels mm^-1^	θ_max_ = 25.4°, θ_min_ = 3.3°
ω scans	*h* = −6→5
Absorption correction: multi-scan (REQAB; Jacobson, 1998)	*k* = −15→15
*T*_min_ = 0.897, *T*_max_ = 0.946	*l* = −19→19
9082 measured reflections	

### Refinement

**Table:**

Refinement on *F*^2^	Primary atom site location: structure-invariant direct methods
Least-squares matrix: full	Secondary atom site location: difference Fourier map
*R*[*F*^2^ > 2σ(*F*^2^)] = 0.073	Hydrogen site location: inferred from neighbouring sites
*wR*(*F*^2^) = 0.213	H atoms treated by a mixture of independent and constrained refinement
*S* = 1.10	*w* = 1/[σ^2^(*F*_o_^2^) + (0.0999*P*)^2^ + 0.2158*P*] where *P* = (*F*_o_^2^ + 2*F*_c_^2^)/3
3380 reflections	(Δ/σ)_max_ < 0.001
231 parameters	Δρ_max_ = 0.40 e Å^−^^3^
5 restraints	Δρ_min_ = −0.37 e Å^−^^3^

### Special details

**Table:**

Geometry. All e.s.d.'s (except the e.s.d. in the dihedral angle between two l.s. planes) are estimated using the full covariance matrix. The cell e.s.d.'s are taken into account individually in the estimation of e.s.d.'s in distances, angles and torsion angles; correlations between e.s.d.'s in cell parameters are only used when they are defined by crystal symmetry. An approximate (isotropic) treatment of cell e.s.d.'s is used for estimating e.s.d.'s involving l.s. planes.
Refinement. Refinement of *F*^2^ against ALL reflections. The weighted *R*-factor *wR* and goodness of fit *S* are based on *F*^2^, conventional *R*-factors *R* are based on *F*, with *F* set to zero for negative *F*^2^. The threshold expression of *F*^2^ > σ(*F*^2^) is used only for calculating *R*-factors(gt) *etc*. and is not relevant to the choice of reflections for refinement. *R*-factors based on *F*^2^ are statistically about twice as large as those based on *F*, and *R*- factors based on ALL data will be even larger.

### Fractional atomic coordinates and isotropic or equivalent isotropic displacement parameters (Å^2^)

**Table:**

	*x*	*y*	*z*	*U*_iso_*/*U*_eq_	Occ. (<1)
Cl1	1.2590 (3)	0.91744 (12)	0.10142 (11)	0.1191 (6)	
O1	0.3603 (6)	0.3847 (3)	0.09779 (16)	0.0827 (9)	
O2	0.4837 (5)	0.2561 (2)	0.23066 (16)	0.0671 (7)	
O3	0.2050 (4)	0.4200 (2)	0.31239 (15)	0.0632 (7)	
N1	0.6503 (5)	0.4156 (2)	0.27693 (15)	0.0447 (7)	
H1	0.8097	0.4099	0.2946	0.054*	
C1	0.4725 (7)	0.3599 (3)	0.1656 (2)	0.0535 (9)	
C2	0.6145 (6)	0.4408 (3)	0.18535 (19)	0.0469 (8)	
C3	0.7106 (7)	0.5258 (3)	0.1182 (2)	0.0512 (8)	
H3	0.6848	0.5267	0.0622	0.061*	
C4	0.8503 (7)	0.6182 (3)	0.1178 (2)	0.0502 (8)	
C5	1.0086 (9)	0.6722 (4)	0.0447 (2)	0.0761 (12)	
H5	1.0296	0.6466	−0.0025	0.091*	
C6	1.1369 (10)	0.7627 (4)	0.0391 (3)	0.0880 (14)	
H6	1.2450	0.7968	−0.0107	0.106*	
C7	1.1039 (8)	0.8020 (3)	0.1076 (3)	0.0712 (11)	
C8	0.9483 (10)	0.7509 (4)	0.1812 (3)	0.0811 (13)	
H8	0.9275	0.7775	0.2279	0.097*	
C9	0.8221 (9)	0.6602 (3)	0.1863 (2)	0.0709 (11)	
H9	0.7157	0.6261	0.2366	0.085*	
C10	0.4399 (6)	0.4007 (3)	0.33605 (19)	0.0448 (8)	
C11	0.5016 (6)	0.3597 (3)	0.43160 (19)	0.0476 (8)	
C12	0.7329 (8)	0.2865 (3)	0.4664 (2)	0.0645 (10)	
H12	0.8642	0.2623	0.4298	0.077*	
C13	0.7701 (10)	0.2483 (4)	0.5579 (3)	0.0851 (14)	
H13	0.9260	0.1981	0.5825	0.102*	
C14	0.5761 (11)	0.2853 (5)	0.6111 (3)	0.0839 (14)	
H14	0.6008	0.2595	0.6718	0.101*	
C15	0.3528 (11)	0.3577 (5)	0.5768 (3)	0.0859 (13)	
H15	0.2245	0.3834	0.6133	0.103*	
C16	0.3112 (8)	0.3944 (4)	0.4881 (2)	0.0680 (11)	
H16	0.1523	0.4436	0.4652	0.082*	
C17	0.3195 (10)	0.1773 (4)	0.2212 (3)	0.0856 (14)	
H17A	0.3550	0.1721	0.1631	0.103*	
H17B	0.1285	0.2063	0.2268	0.103*	
C18	0.3886 (8)	0.0619 (4)	0.2908 (3)	0.0781 (12)	
H18	0.275 (6)	0.012 (3)	0.281 (2)	0.073 (11)*	
C19	0.669 (2)	0.0005 (11)	0.3151 (8)	0.107 (2)*	0.50
H19A	0.7628	0.0472	0.3336	0.160*	0.50
H19B	0.7638	−0.0122	0.2646	0.160*	0.50
H19C	0.6603	−0.0729	0.3629	0.160*	0.50
C19'	0.662 (2)	0.0186 (11)	0.2528 (8)	0.107 (2)*	0.50
H19D	0.7984	0.0599	0.2579	0.160*	0.50
H19E	0.6450	0.0320	0.1913	0.160*	0.50
H19F	0.7126	−0.0632	0.2853	0.160*	0.50
C20	0.262 (2)	0.0812 (11)	0.3758 (7)	0.101 (2)*	0.50
H20A	0.2481	0.0073	0.4224	0.152*	0.50
H20B	0.0847	0.1264	0.3638	0.152*	0.50
H20C	0.3748	0.1218	0.3937	0.152*	0.50
C20'	0.422 (2)	0.0469 (11)	0.3842 (7)	0.101 (2)*	0.50
H20D	0.4701	−0.0342	0.4191	0.152*	0.50
H20E	0.2546	0.0770	0.4076	0.152*	0.50
H20F	0.5625	0.0883	0.3862	0.152*	0.50

### Atomic displacement parameters (Å^2^)

**Table:**

	*U*^11^	*U*^22^	*U*^33^	*U*^12^	*U*^13^	*U*^23^
Cl1	0.1242 (12)	0.0830 (9)	0.1589 (14)	−0.0548 (8)	−0.0204 (10)	−0.0333 (9)
O1	0.116 (2)	0.088 (2)	0.0517 (14)	−0.0472 (18)	−0.0216 (15)	−0.0154 (14)
O2	0.0817 (18)	0.0547 (16)	0.0676 (15)	−0.0262 (13)	−0.0179 (13)	−0.0148 (13)
O3	0.0374 (13)	0.0924 (19)	0.0553 (13)	−0.0180 (12)	−0.0059 (10)	−0.0178 (13)
N1	0.0384 (13)	0.0568 (17)	0.0390 (13)	−0.0168 (12)	−0.0038 (11)	−0.0129 (12)
C1	0.057 (2)	0.059 (2)	0.0469 (18)	−0.0184 (17)	−0.0037 (16)	−0.0170 (17)
C2	0.0462 (18)	0.056 (2)	0.0393 (16)	−0.0155 (15)	−0.0004 (13)	−0.0148 (15)
C3	0.057 (2)	0.059 (2)	0.0412 (16)	−0.0174 (17)	−0.0015 (14)	−0.0190 (16)
C4	0.0516 (19)	0.052 (2)	0.0432 (16)	−0.0101 (16)	−0.0044 (14)	−0.0117 (15)
C5	0.095 (3)	0.078 (3)	0.059 (2)	−0.039 (2)	0.018 (2)	−0.024 (2)
C6	0.096 (3)	0.086 (3)	0.084 (3)	−0.051 (3)	0.024 (2)	−0.025 (3)
C7	0.066 (2)	0.056 (2)	0.090 (3)	−0.018 (2)	−0.016 (2)	−0.017 (2)
C8	0.117 (4)	0.062 (3)	0.072 (3)	−0.030 (3)	−0.012 (3)	−0.024 (2)
C9	0.098 (3)	0.063 (2)	0.056 (2)	−0.034 (2)	0.005 (2)	−0.0187 (18)
C10	0.0439 (18)	0.0495 (19)	0.0404 (16)	−0.0147 (15)	−0.0017 (14)	−0.0124 (14)
C11	0.0487 (19)	0.051 (2)	0.0432 (16)	−0.0219 (16)	−0.0031 (15)	−0.0103 (15)
C12	0.061 (2)	0.069 (3)	0.0494 (18)	−0.0140 (19)	−0.0051 (17)	−0.0045 (18)
C13	0.082 (3)	0.087 (3)	0.066 (2)	−0.024 (3)	−0.027 (2)	0.004 (2)
C14	0.105 (4)	0.103 (4)	0.047 (2)	−0.054 (3)	−0.002 (2)	−0.016 (2)
C15	0.105 (4)	0.103 (4)	0.055 (2)	−0.029 (3)	0.004 (2)	−0.031 (2)
C16	0.069 (2)	0.085 (3)	0.053 (2)	−0.015 (2)	0.0020 (18)	−0.028 (2)
C17	0.104 (3)	0.068 (3)	0.092 (3)	−0.044 (3)	−0.022 (3)	−0.019 (2)
C18	0.067 (3)	0.060 (3)	0.112 (3)	−0.020 (2)	−0.014 (2)	−0.029 (2)

### Geometric parameters (Å, °)

**Table:**

Cl1—C7	1.734 (4)	C13—C14	1.371 (7)
O1—C1	1.200 (4)	C13—H13	0.9300
O2—C1	1.328 (4)	C14—C15	1.331 (7)
O2—C17	1.458 (4)	C14—H14	0.9300
O3—C10	1.232 (4)	C15—C16	1.365 (5)
N1—C10	1.341 (4)	C15—H15	0.9300
N1—C2	1.426 (4)	C16—H16	0.9300
N1—H1	0.8600	C17—C18	1.470 (6)
C1—C2	1.485 (5)	C17—H17A	0.9700
C2—C3	1.327 (4)	C17—H17B	0.9700
C3—C4	1.459 (5)	C18—C20'	1.483 (10)
C3—H3	0.9300	C18—C19	1.490 (10)
C4—C5	1.375 (5)	C18—C19'	1.540 (11)
C4—C9	1.390 (5)	C18—C20	1.546 (10)
C5—C6	1.377 (6)	C18—H18	0.99 (3)
C5—H5	0.9300	C19—H19A	0.9600
C6—C7	1.367 (6)	C19—H19B	0.9600
C6—H6	0.9300	C19—H19C	0.9600
C7—C8	1.363 (6)	C19'—H19D	0.9600
C8—C9	1.375 (5)	C19'—H19E	0.9600
C8—H8	0.9300	C19'—H19F	0.9600
C9—H9	0.9300	C20—H20A	0.9600
C10—C11	1.489 (4)	C20—H20B	0.9600
C11—C12	1.367 (5)	C20—H20C	0.9600
C11—C16	1.386 (5)	C20'—H20D	0.9600
C12—C13	1.404 (5)	C20'—H20E	0.9600
C12—H12	0.9300	C20'—H20F	0.9600
			
C1—O2—C17	116.2 (3)	C13—C14—H14	119.7
C10—N1—C2	121.2 (2)	C14—C15—C16	120.3 (4)
C10—N1—H1	119.4	C14—C15—H15	119.8
C2—N1—H1	119.4	C16—C15—H15	119.8
O1—C1—O2	123.0 (3)	C15—C16—C11	121.1 (4)
O1—C1—C2	123.9 (3)	C15—C16—H16	119.4
O2—C1—C2	113.1 (3)	C11—C16—H16	119.4
C3—C2—N1	124.9 (3)	O2—C17—C18	108.7 (3)
C3—C2—C1	118.8 (3)	O2—C17—H17A	110.0
N1—C2—C1	116.2 (3)	C18—C17—H17A	110.0
C2—C3—C4	130.6 (3)	O2—C17—H17B	110.0
C2—C3—H3	114.7	C18—C17—H17B	110.0
C4—C3—H3	114.7	H17A—C17—H17B	108.3
C5—C4—C9	116.9 (3)	C17—C18—C20'	122.0 (6)
C5—C4—C3	119.6 (3)	C17—C18—C19	125.4 (6)
C9—C4—C3	123.4 (3)	C20'—C18—C19	72.4 (7)
C4—C5—C6	122.2 (4)	C17—C18—C19'	100.6 (6)
C4—C5—H5	118.9	C20'—C18—C19'	108.9 (7)
C6—C5—H5	118.9	C17—C18—C20	102.6 (6)
C7—C6—C5	119.3 (4)	C19—C18—C20	104.4 (7)
C7—C6—H6	120.3	C19'—C18—C20	140.7 (7)
C5—C6—H6	120.3	C17—C18—H18	105 (2)
C8—C7—C6	120.3 (4)	C20'—C18—H18	117 (2)
C8—C7—Cl1	119.5 (3)	C19—C18—H18	112 (2)
C6—C7—Cl1	120.2 (3)	C19'—C18—H18	99 (2)
C7—C8—C9	119.9 (4)	C20—C18—H18	105 (2)
C7—C8—H8	120.0	C18—C19—H19A	109.5
C9—C8—H8	120.0	C18—C19—H19B	109.5
C8—C9—C4	121.4 (4)	C18—C19—H19C	109.5
C8—C9—H9	119.3	C18—C19'—H19D	109.5
C4—C9—H9	119.3	C18—C19'—H19E	109.5
O3—C10—N1	121.4 (3)	H19D—C19'—H19E	109.5
O3—C10—C11	121.2 (3)	C18—C19'—H19F	109.5
N1—C10—C11	117.3 (3)	H19D—C19'—H19F	109.5
C12—C11—C16	118.7 (3)	H19E—C19'—H19F	109.5
C12—C11—C10	123.0 (3)	C18—C20—H20A	109.5
C16—C11—C10	118.3 (3)	C18—C20—H20B	109.5
C11—C12—C13	119.3 (4)	C18—C20—H20C	109.5
C11—C12—H12	120.4	C18—C20'—H20D	109.5
C13—C12—H12	120.4	C18—C20'—H20E	109.5
C14—C13—C12	119.9 (4)	H20D—C20'—H20E	109.5
C14—C13—H13	120.1	C18—C20'—H20F	109.5
C12—C13—H13	120.1	H20D—C20'—H20F	109.5
C15—C14—C13	120.6 (4)	H20E—C20'—H20F	109.5
C15—C14—H14	119.7		
			
C17—O2—C1—O1	−8.2 (5)	C3—C4—C9—C8	−177.0 (4)
C17—O2—C1—C2	172.3 (3)	C2—N1—C10—O3	−7.7 (5)
C10—N1—C2—C3	130.9 (4)	C2—N1—C10—C11	172.3 (3)
C10—N1—C2—C1	−52.9 (4)	O3—C10—C11—C12	147.3 (4)
O1—C1—C2—C3	−25.4 (5)	N1—C10—C11—C12	−32.7 (5)
O2—C1—C2—C3	154.1 (3)	O3—C10—C11—C16	−30.4 (5)
O1—C1—C2—N1	158.2 (3)	N1—C10—C11—C16	149.6 (3)
O2—C1—C2—N1	−22.3 (4)	C16—C11—C12—C13	0.3 (5)
N1—C2—C3—C4	−5.6 (6)	C10—C11—C12—C13	−177.4 (3)
C1—C2—C3—C4	178.3 (3)	C11—C12—C13—C14	−0.5 (6)
C2—C3—C4—C5	158.4 (4)	C12—C13—C14—C15	−0.4 (7)
C2—C3—C4—C9	−25.0 (6)	C13—C14—C15—C16	1.3 (7)
C9—C4—C5—C6	0.7 (6)	C14—C15—C16—C11	−1.5 (7)
C3—C4—C5—C6	177.5 (4)	C12—C11—C16—C15	0.6 (6)
C4—C5—C6—C7	−1.0 (7)	C10—C11—C16—C15	178.5 (3)
C5—C6—C7—C8	0.8 (7)	C1—O2—C17—C18	171.3 (3)
C5—C6—C7—Cl1	−178.9 (4)	O2—C17—C18—C20'	43.9 (8)
C6—C7—C8—C9	−0.5 (7)	O2—C17—C18—C19	−46.5 (8)
Cl1—C7—C8—C9	179.3 (3)	O2—C17—C18—C19'	−76.4 (6)
C7—C8—C9—C4	0.3 (7)	O2—C17—C18—C20	71.7 (6)
C5—C4—C9—C8	−0.4 (6)		

### Hydrogen-bond geometry (Å, °)

**Table:**

*D*—H···*A*	*D*—H	H···*A*	*D*···*A*	*D*—H···*A*
C3—H3···O1^i^	0.93	2.43	3.299 (4)	155
N1—H1···O3^ii^	0.86	2.07	2.916 (3)	169
C9—H9···N1	0.93	2.55	3.103 (4)	119

Symmetry codes: (i) −*x*+1, −*y*+1, −*z*; (ii) *x*+1, *y*, *z*.
